# Electrophysiological and behavioral correlates of cannabis use disorder

**DOI:** 10.3758/s13415-022-01016-w

**Published:** 2022-06-13

**Authors:** Théo Andriot, Patrick Ohnmacht, Patrik Vuilleumier, Gabriel Thorens, Yasser Khazaal, Nathalie Ginovart, Tomas Ros

**Affiliations:** 1 Department of Basic Neurosciences, University of Geneva, Geneva, Switzerland; 2 Institut de Biologie Paris Seine, Neuroscience Paris Seine, Inserm U1130, CNRS UMR8246, Sorbonne Université, Paris, France; 3 Department of Psychiatry, University of Geneva, Geneva, Switzerland; 4 Center For Biomedical Imaging, Lausanne, Geneva, Switzerland; 5 Department of Psychiatry, Centre Hospitalier Universitaire Vaudois, Lausanne, Switzerland

**Keywords:** Cannabis use disorder, Alpha oscillation, Cortical activation, Electroencephalography, Attentional performance

## Abstract

Current research indicates deficits in cognitive function together with widespread changes in brain activity following long-term cannabis use. In particular, cannabis use has been associated with excessive spectral power of the alpha rhythm (8–12 Hz), which is also known to be modulated during attentional states. Recent neuroimaging studies have linked heavy cannabis use with structural and metabolic changes in the brain; however, the functional consequences of these changes are still not fully characterized. This study investigated the electrophysiological and behavioral correlates of cannabis dependence by comparing patients with a cannabis use disorder (CUD; N = 24) with cannabis nonuser controls (N = 24), using resting state electroencephalogram (EEG) source-imaging. In addition to evaluating mean differences between groups, we also explored whether particular EEG patterns were associated with individual cognitive-behavioral measures. First, we replicated historical findings of elevated levels of (relative) alpha rhythm in CUD patients compared with controls and located these abnormalities to mainly prefrontal cortical regions. Importantly, we observed a significant negative correlation between alpha spectral power in several cortical regions and individual attentional performance in the Go/NoGo task. Because such relationship was absent in the nonuser control group, our results suggest that reduced prefrontal cortical activation (indexed by increased relative alpha power) could be partly responsible for the reported cognitive impairments in CUD. Our findings support the use of electroencephalography as a noninvasive and cost-effective tool for biomarker discovery in substance abuse and have the potential of directly informing future intervention strategies.

**Supplementary Information:**

The online version contains supplementary material available at 10.3758/s13415-022-01016-w.

## Introduction

Delta-9-tetrahydrocannabinol (THC), the principal constituent of cannabis, binds to cannabinoid-type 1 receptors (CB1R), which are most abundant in the frontal cortex, hippocampus, and cerebellum (Battisti et al., 2010). Repeated THC exposure alter CB1R functioning, with consequences on neural processes implemented in these brain regions. Consequently, cannabis users exhibits drug-induced synaptic plasticity on brain circuitry and changes in neuronal physiology underlying maladaptive behaviors (Hyman & Malenka, 2001; Kalivas & O'Brien, 2008; Solowij et al., 2009).

Animal studies report that THC disrupts the regulation of the endogenous cannabinoid system leading to pathological changes in brain structure and function, including network connectivity (Batalla et al., 2013). By binding on CB1R, THC also indirectly induces functional alterations of dopamine (DA) pathways. Within this framework, antagonistic interactions between CB1R and dopamine D2/3 receptors (D2/3R) lead to DA hyperactivation and hypersensitization of D2/3R in the striatum (Ginovart et al., 2012). Brain structural changes include decreases of cerebrospinal fluid and grey matter density (Block et al., 2000; Matochik et al., 2005), while functionally there are decreases of cerebral blood flow and downregulation of CB1R (Hirvonen et al., 2012; Lundqist et al., 2001). This is compatible with several human neuroimaging studies reporting a pattern of brain regions dysfunction, such as prefrontal, parietal, and limbic cortices, associated with worse outcome, indicating impacts of long-term cannabis use (Goldstein & Volkow, 2011; Lundqvist, 2005). Although *results* remain *inconsistent* between studies, likely due to sociodemographic and drug use differences (Batalla et al., 2013). Importantly, the relationship between cannabis-related changes and their impact on cognitive performance remains to be elucidated.

To distangle this relationship and examine the subtle dysfunction characteristics of cannabis exposure, electroencephalography (EEG) provides a valuable electrophysiological method for noninvasive exploration of high temporal resolution (i.e., millisecond) electrocortical fluctuations (Norberg et al., 2012).

In electrocortical profiles recorded by EEG,previous studies showed that cannabis exposure is associated with a unique topography of EEG activity, which includes an increased absolute and relative power of alpha “slow waves” over the bilateral frontal cortex (Struve et al., 1999; Struve et al., 2003). Importantly, alpha oscillations are known to be modulated by attention and display a negative correlation with cortical activation and metabolism (Conner et al., 2011).

At the behavioral level, cannabis use has been associated with a wide range of cognitive and executive deficits (Accordino et al., 2006; Solowij & Pesa, 2010). Impairments in attention are most commonly observed following cannabis abuse (Lundqvist, 2005), whereas personality traits of impulsivity are widespread among chronic users (Ersche et al., 2010). A neurophysiological origin for these impairments is supported by noninvasive imaging studies showing aberrant activation of intrinsic brain networks (Goldstein & Volkow, 2011). Other studies showed that increases in trial-by-trial alpha power predict failures to inhibit prepotent motor responses during a response inhibition task (Mazaheri et al., 2009; Mazaheri et al., 2011).

Elsewhere, several studies have linked cannabis use with altered DA function and brain connectivity, leading to cognitive problems (Bloomfield et al., 2014; Coullaut-Valera et al., 2014). However, because the neurocognitive substrates of these changes remain poorly understood, a clearer investigation of the mechanisms associated with repeated cannabis use is needed. Notably, we sought to directly investigate potential associations between patients’ attentional deficits and abnormal EEG activity.

In the current study, we investigated in tandem the EEG and behavioral correlates of cannabis use disorder (CUD). This was achieved by examining the potential EEG abnormalities of CUD patients compared with nonusers and comparing measures of cognitive performance in the Go/NoGo task between CUD patients and nonusers controls.

We first hypothesized that patients with CUD exhibit significant changes in EEG spectral profile and attentional deficits compared with cannabis nonuser controls and that these brain-behavioral measures may be cross-correlated. Specifically, based on earlier work (Accordino et al., 2006; Kalivas & O'Brien, 2008; Lundqist et al., 2001), we expected CUD patients to demonstrate elevated EEG alpha power and deficient attentional performance (i.e., lower accuracy).

## Materials and Methods

### Participants

A total of 48 participants were recruited, including 24 patients with a diagnosis of CUD (mean age: 26; standard deviation [SD]: 5.39; 10 women, 14 men) and 24 age- and sex-matched healthy controls without history of cannabis use (mean age: 24.54; SD: 4.4; 11 women, 13 men).

A clinical screening interview was conducted with each potential participant to determine eligibility for the study. Participants with past or current psychiatric or neurological disorders, past or current clinically significant medical condition and central nervous system disorder, addictive disorders (other than cannabis use in patients, and except tobacco), or current psychotropic treatment were excluded.

Control participants followed the same screening interview as patients and were selected to match cannabis users on key demographics (i.e., sex, age, and educational experience). Demographics parameters differences between CUD patients and nonuser controls were not significant except degree of cannabis use as expected (Supplementary Table 1). All participants gave their written, informed consent before participating in the study, which was approved by Geneva Ethics Committee and accorded with the declaration of Helsinki.

### Screening

#### Cannabis consumption

To qualify as CUD, diagnosis was assessed using the DSM-5 manual, which includes 11 diagnostic criteria and defines this disorder by the presence of at least 2 of them occurring in a 12-month period. The severity of cannabis consumption was evaluated by two variables: the frequency of cannabis consumption per week and the time to smoke a quantity of 1 g of cannabis. This is more accurate than subjective recall of the number of cannabis cigarettes, given the variability in dose and self-reported use (Bloomfield et al., 2014).

To characterize chronic use, we generally lack reliable information on the doses of THC that is commonly used by regular cannabis users (Norberg et al., 2012). As a consequence, epidemiological studies have usually defined “heavy” or “regular” cannabis use as daily or near daily use, because this pattern of continued use over years predicts increased risks of adverse health effects (Hall & Pacula, 2002). Accordingly, in our study we applied a combination of criteria, including weekly consumption and age at first use, to qualify participants as regular cannabis users. Furthermore, in our sample, all patients reported consuming cannabis exclusively through cigarettes.

Detailed drug histories were obtained from participants using questionnaires to avoid other forms of addiction and comorbidities. Patients were assessed using the Alcohol, Smoking, and Substance Involvement Screening Test (ASSIST), a short screening questionnaire to evaluate the use of different substances (tobacco products, alcohol, cannabis, cocaine, amphetamine-type stimulants, sedatives and sleeping pills, hallucinogens, inhalants, opioids, and “other drugs”) and consequences associated (Humeniuk et al., 2008; Khan et al., 2011). Finally, CUD participants were asked to stop their cannabis use 24 h before the screening to preclude any possible acute influence on measures.

#### Go/NoGo task

**Fig. 1:**
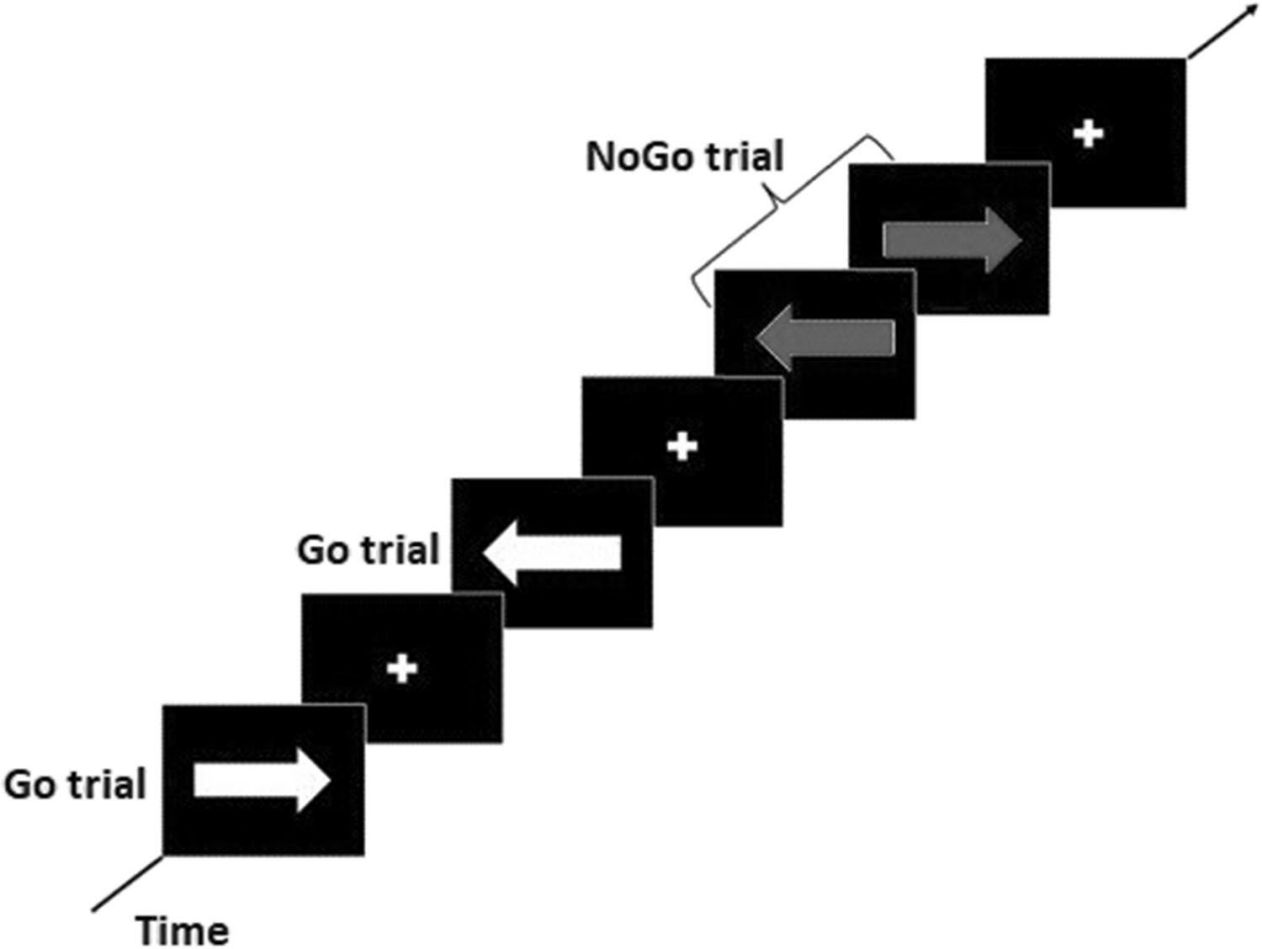
Schematic display of the Go NoGo task paradigm (Gan et al., 2014)

The Go/NoGo is a computerized task used to study sustained attention and inhibitory control as hallmarks of executive functioning, which is generally altered in substance-abuse disorders (Verbruggen & Logan, 2008). This task measures the capacity to inhibit a pre-potent response (i.e., dominant or automatic motor response). It consists in left- or right- pointing arrows appearing briefly on the screen, participants must select the corresponding on the keyboard as fast as possible (Go condition). Randomly on 20% of trials, the arrow color changes to blue, this represents the NoGo condition in which the motor response must be inhibited Fig. 1.

The parameters of the task were set as follows:

- Maximal reaction time: 1.0 s
- Intertrial interval: 0.25 s
- Initial signal delay: 0.250 s
- Numbers of experiment blocks: 2
- Delay between blocks: 30 s, with feedback on the performance of each block.

The random list for signal presentation was organized with 10 Go stimuli and 2 NoGo stimuli (1/6 of the trials were NoGo trials). The task included 1 practice block, which contains 24 trials with 20 Go and 4 NoGo stimuli, and 2 experimental blocks in which design was repeated 20 times. At the end of the task, the subject’s performance parameters were recorded, as the numbers of Go and No-Go responses for each condition: correct, incorrect on a NoGo, incorrect on a Go, and missed.

Results were analysed using the framework of Signal Detection theory (Macmillan & Creelman, 2005), which attributes responses to a combination of sensitivity (as detecting a signal, i.e., Go) and specificity (i.e., NoGo). Here, accuracy is represented by the Hit rate (success rate), i.e., the proportion of correct responses on Go trials (conversely known as omission errors) and was used as an index of attentional performance (Li et al., 2006; Saunders et al., 2008). The proportion of incorrect responses on NoGo trials (i.e., commission error) was used as an index of inhibitory control (Li et al., 2006; Macmillan & Creelman, 2005).

### EEG recording and processing

Quantitative EEGs were obtained after subjects passed screening examination. A multichannel EEG cap was used to measure whole-scalp activity in baseline recording, consisted of resting state measurement of 3 minutes under eyes open and closed conditions. The scalp signals were recorded using a 19 Ag/AgCl electrodes cap (Electro-cap International, Inc. www.electro-cap.com) according to the 10-20 international system. The ground electrode was placed on the scalp equidistant between Fpz and Fz. Electrical signals were amplified with the 21-channel Mitsar EEG system (Mitsar-201, CE0537, Mitsar, Ltd. http://www.mitsar-medical.com), and all electrode impedances was set below 5 kOhm. For online recording, electrodes were referenced to linked earlobes, and then the common average reference was calculated offline before further analysis.EEG data were continuously recorded at a sampling rate of 250 Hz and then filtered with an offline bandpass filter of 0.5-50 Hz.

For offline processing, all EEG data were imported into the Matlab toolbox EEGLAB v12 (http://sccn.ucsd.edu/eeglab/). We removed stereotypical eye movement artifacts, such as saccades or blinking, using Infomax ICA decomposition (Jung et al., 2000). This provides a good artifact separation performance for most artifact types whilst ensuring minimum information loss (Inuso et al., 2007). Recordings were further cleaned with an automated z-score based method, using the FASTER plugin (Nolan et al., 2010), rejecting 1-second epochs that deviated from the mean by more than 1.7 standard deviations.

### Source-space measures of EEG activity

Artifact-free EEG data were processed in Matlab with the Brainstorm Toolbox (http://neuroimage.usc.edu/brainstorm/). In line with previous approaches using a similar EEG setup in clinical populations (Tokariev et al., 2019), we first computed a head model of the cortex surface for each EEG recording using overlapping spheres (OpenMEEG) and then estimated unconstrained cortical sources using the minimum-norm sLORETA algorithm implemented in Brainstorm (Cosandier-Rimélé et al., 2017). To normalize sources across participants, we projected (warped) the sources from each participant onto the MNI/Colin27 template brain (Holmes et al., 1998). The 15,000 voxel source-space was then divided into 68 cortical regions-of-interest (ROIs) according to the Desikan–Killiany neuroanatomical atlas (Desikan et al., 2006). Temporal source-activities across all the voxels in each ROI were then averaged and band-pass filtered in the following 4 frequency bands: delta 1-4 Hz, theta 4-8 Hz, alpha 8-12 Hz, beta 13-20 Hz. For every subject, each frequency band was quantified in Brainstorm to examine differences in spectral power between the patient and control groups.

#### Spectral power

Spectral subdivision into the basic EEG frequency bands was estimated with a standard FFT approach using Welch’s method (Matlab pwelch() function) and a Hanning windowing function (4 second epoch, 50% overlap). Based on the total number of collected and averaged epochs per subject, quantitative estimates of Absolute Power (amplitude, signal strength) was computed, and percent Relative Power (i.e., % power, amount, abundance) was calculated as the ratio of the mean power in a specific EEG band and the broadband power (1-45 Hz).

### Statistical analyses

Source-space (voxel-wise) of band-limited surface spectral power were export by using the Brainstorm Toolbox. Statistical comparisons between CUD patients and controls were performed using Statistical Parametric Mapping toolbox (SPM12) via independent two-tailed t-tests with a *p* < 0.05 threshold across the four EEG bands. Individual attentional performance was measured by performance on the Go/NoGo task, using the Hit rate variable. Data from CUD group was subsequently used in a between-subject regression analysis (with a *p* < 0.05 threshold) using the SPM toolbox.

Between-group comparisons in inhibitory control (commission errors) and attention (hit rate) in the Go/NoGo task were performed by using independent two-tailed t-tests with a probability of type I error (α) = 0.05. One control subject outlier was excluded from the analysis, because his EEG power measure was 2 standard deviations above the sample’s mean.

Considering the previous studies reporting alpha band abnormalities in CUD (Struve et al., 1999; Struve et al., 2003), our main hypotheses were based on this band and hence multiple comparisons involving alpha spectral power were performed without statistical correction. Comparisons involving delta, theta, and beta bands also were uncorrected but may be considered exploratory.

## Results

### Group spectral power in patients compared with healthy controls

**Fig. 2:**
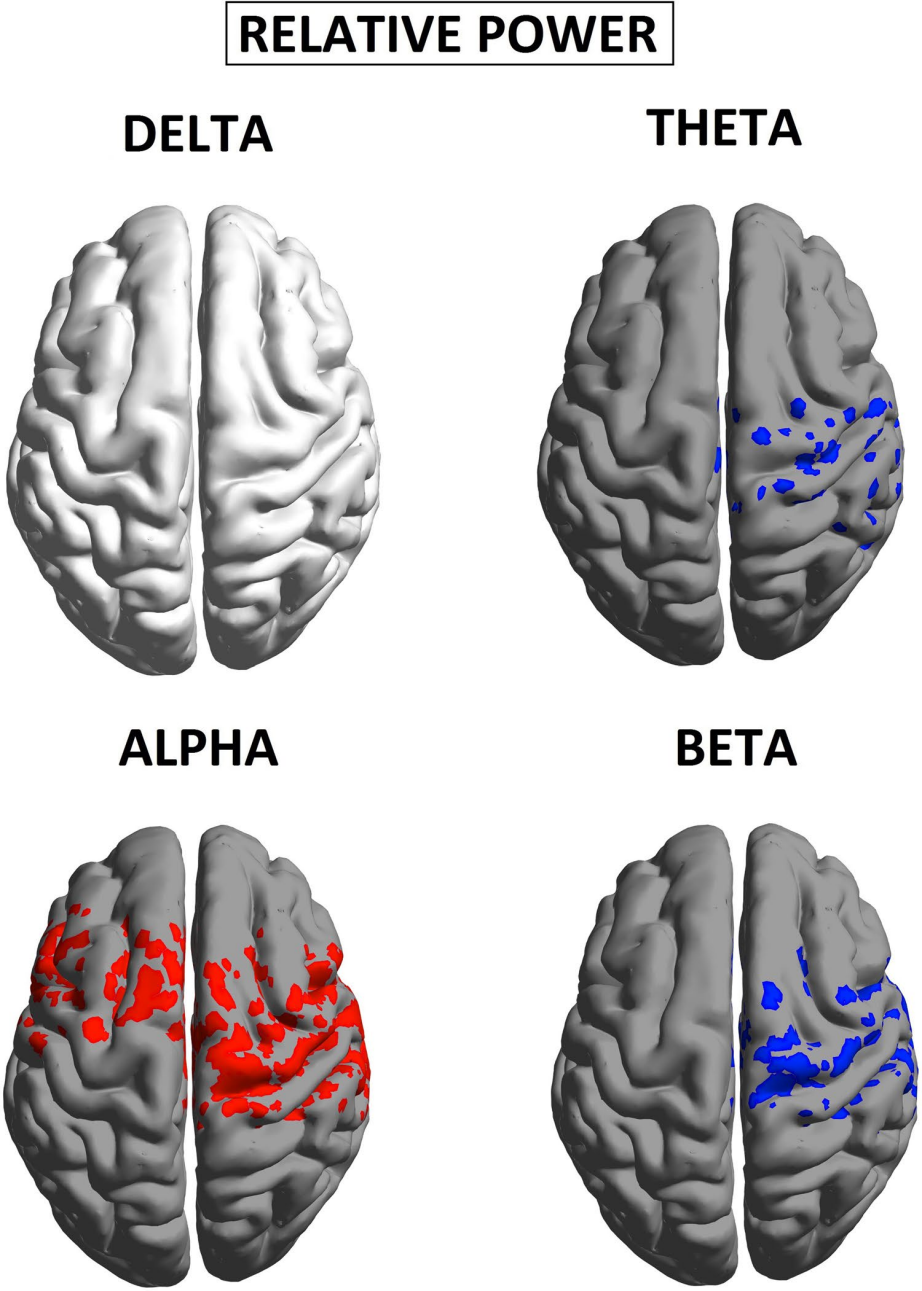
Relative spectral power, during eyes closed. *p* values of statistical differences in BrainNet viewer source-space between CUD patients and nonuser controls. *Red* indicates greater power for patients (*p* < 0.05); *blue* indicates greater power for controls (*p* < 0.05)

**Fig. 3:**
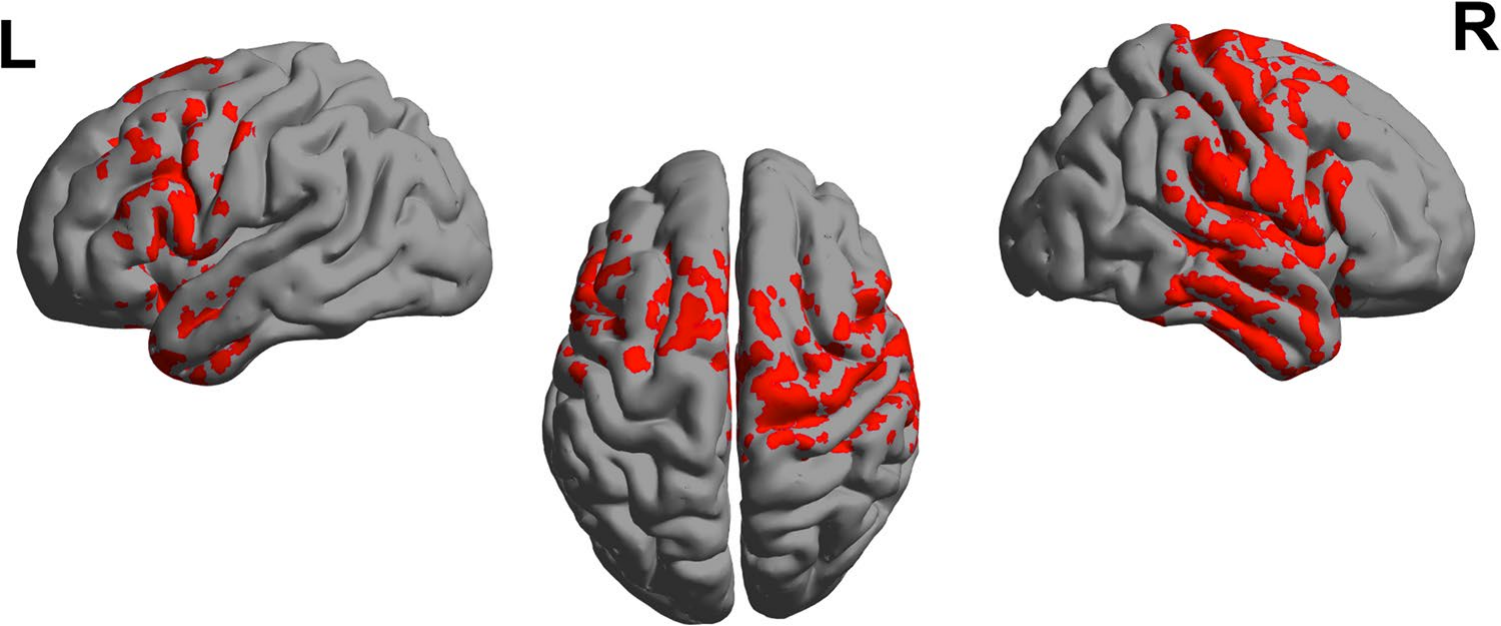
Relative spectral power of alpha band, during eyes closed. *p* values of statistical differences in BrainNet viewer source-space between CUD patients and controls. *Red* indicates greater power for patients (*p* < 0.05)

An overview of the results obtained is provided in Table 1, as specifically shown in Fig 2 resting state EEG showed significant increases in spectral power for CUD patients compared with controls across all four frequency bands, albeit this effect was most distributed in the alpha band (Fig 2). To improve specificity, we tested for differences in relative (%) power, often used to normalize spectra under a constant value of broadband (1-40 Hz) power, and reflecting the degree of spectral slowing (i.e., greater relative power in lower frequencies) or spectral acceleration (i.e., greater relative power in higher frequencies). As shown in Fig. 2, patients demonstrated regions of anatomically selective excess of slow-waves amplitude (alpha), in line with previous research (Kalivas & O'Brien, 2008). Specifically, relative alpha power was significantly more elevated in the cortex of patients relative compared with controls (Fig 3), with a statistical threshold of *p* < 0.05 at a t-values of t >1.67.

**Table 1:** Overview of the statistical analyses and results obtained on the comparison between CUD patients and healthy controls. Comparisons of delta, theta, and beta bands were only exploratory

EEG and behavioral results			Brain-behavioral correlates	Conjunction analysis
Relative spectral power	Delta	*n.s.*		
	Theta	t = −1.99; *p* < 0.05 in temporal lobes		
	Beta	t = −1.87; *p* < 0.05 in temporal lobes		
	Alpha	t = 2.11; *p* < 0.05 in sensorimotor cortex	t = −2.40; *p* < 0.05 Negative correlation over sensorimotor cortex	t = 1.50; *p* < 0.05 over Statistical overlap on left sensorimotor and temporal regions
Go/NoGo task	Hit rate	*n.s.*		
	Commission errors	*n.s.*		

*n.s.* not significant

CUD was associated with relative alpha power increases within frontocentral regions, with a maximum in the sensorimotor cortex (t = 2.11, *p* < 0.05). On the other hand, relative theta and beta power were reduced within the temporal lobe (t = −1.99, *p* < 0.05; t = −1.87, *p* < 0.05 respectively). No significant differences were found in the delta band.

### Brain-behavioral correlates

There were no statistical differences in either commission errors (*p* > 0.05) or hit rate (*p* > 0.05) between CUD patients and controls, indicating that inhibitory control and sustained attention were similar between the two groups.

**Fig. 4:**
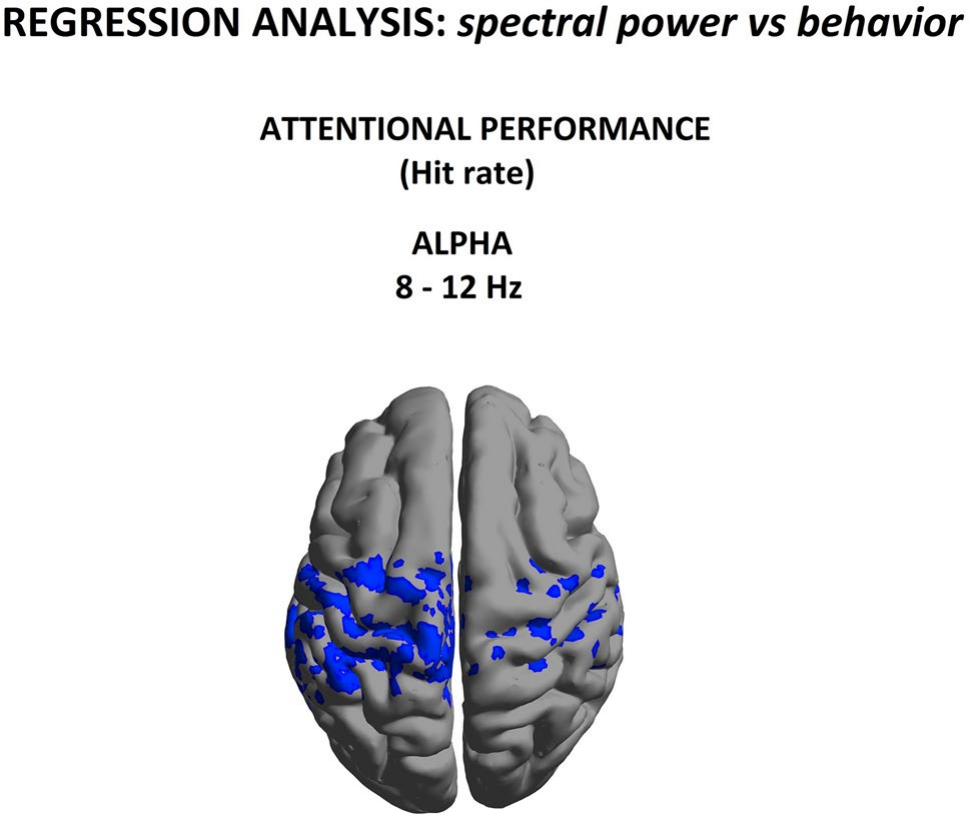
Alpha (8-12 Hz) power as a function of hit rate score in the Go/NoGo task. Regression obtained using data from CUD group only. Blue values indicate statistically significant beta coefficients (*p* < 0.05)

Our group-level analyses indicated anomalous spectral power pattern between patients and control subjects; however, they cannot be used to directly infer any potential links with attentional performance. To disentangle neurobehaviorally specific from nonspecific EEG power in CUD patients, we conducted regression analyses directly testing for any relationships between individual patients’ relative alpha source power and their attentional performance, as indexed by the Hit score (success rate) in the Go/NoGo task. As seen in Fig. 4, a significant negative correlation (t = −2.40, *p* < 0.05) with attentional performance indicates that decrease Hit rate accuracy was predicted by increases in alpha power within a homologous set of nodes in somatosensory and motor Brodmann areas. This is consistent with the literature linking attention with frontal activity in healthy subjects (Mazaheri et al., 2009), and reinforces the idea that the changes in alpha-band may be specifically related to difficulties in spatially directed attentional processing in CUD.

### Conjunction analysis between group spectral power abnormalities and correlates of behavioral deficits

**Fig. 5:**
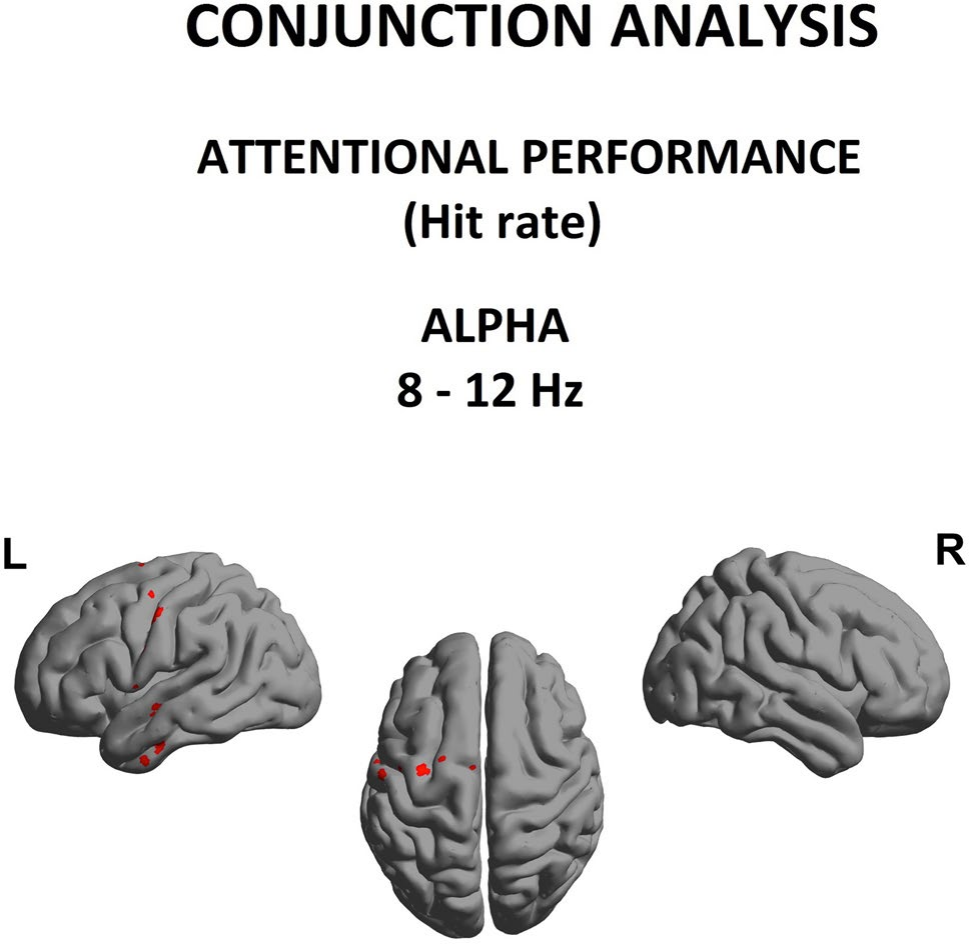
Alpha (8-12 Hz) power as a function of Hit rate score, conjunction analysis using data from control and CUD group

Finally, as shown in Fig. 5, we investigated whether abnormal spectral power seen at the group level was consistent with changes associated with interindividual differences in attentional performance. *In other words, was there evidence for a selective disruption of alpha rhythm in cannabis addiction that impacted attentional function?* Consequently, we performed a conjunction of tests (i.e., global null hypothesis) to identify the overlap, if any, between the statistically-significant (*p* < 0.05) group-level relative alpha power differences (Fig. 2) and the regression between relative alpha power and omission errors (Fig. 4).

Interestingly, we identified a statistically reliable overlap within alpha band and attentional performance in the *left sensorimotor and temporal regions (Brodmann area 4, 6, 20* with maximum (t = 1.50, *p* < 0.05))*.*

## Discussion

The present study focused on the relationship between alpha oscillations and attention in adult cannabis users, using an experimental design with resting EEG and behavioral task conditions. By examining the cortical dynamics of CUD patients using EEG signals, and calculating spectral power profiles, our results indicate that: 1) CUD patients showed increased alpha power over frontal regions compared to controls, and 2) their attentional performance (hit accuracy) inversely correlates with individual levels of frontal alpha power in the sensorimotor cortex (Table 1).

### Differences in EEG spectral power between cannabis users and controls

It has previously been reported that theta/alpha oscillations reflect modulations of neuronal processing within cortical circuits (Klimesch, 1999; Romei et al., 2008; Ros et al., 2010). Specifically, suppression of alpha rhythms results in increased cortical excitability (Ros et al., 2010). Interestingly, excess slow-wave spectral power appears to be a nonspecific marker of brain dysfunction (Ros et al., 2014). For example, patients with attentional-deficit disorder typically present significantly greater magnitudes of theta/alpha rhythms (Koehler et al., 2009), and their normalization (i.e., reduction) during treatment is related to improvements in attention (Clarke et al., 2002; Deiber et al., 2020; Gevensleben et al., 2009). Taken together, these results suggest that cannabis users may suffer from insufficient recruitment of frontal regions with a detrimental impact on attentional capacity.

Specifically, in our study, patients’ cortical activation was reduced compared with control subjects, given that they exhibited an increase in slow-wave alpha power, peaking over the sensorimotor regions (*p* < 0.05). This excess of alpha rhythmicity may be associated with cortical inhibition or reduced information processing (Klimesch, 1999), and its location in frontal regions might be responsible for attentional impairments.

The spectral power results in our study are compatible with previous work reporting an alpha power increases in frontal regions of cannabis users (Struve et al., 1999). On the other hand, our results do not replicate studies showing significant reductions in delta power (Prashad et al., 2018; Struve et al., 1999). These inconsistencies may stem from differences in the characterization of cannabis users, given the potentially wide range of cannabis abuse profiles in these studies.

### Relationship between alpha power and attentional performance

Our analyses correlating EEG with behavioral measures demonstrated a significantly negative correlation between frontal alpha power and hit rates on the Go/NoGo task. This suggests that hypoactive frontal cortical networks could directly underpin attentional deficits in cannabis users. This account is supported by studies reporting a similar inverse correlation between alpha and sensory detection performance in healthy subjects and patients with ADHD (Deiber et al., 2020; Ergenoglu et al., 2004; Mazaheri et al., 2014). Abnormal patterns of electrical oscillatory activity have been repeatedly described in adult ADHD (Poil et al., 2014). In particular, the alpha rhythm (8–12 Hz), known to be modulated during attention, is affected in ADHD, as well as other neurological disorders associated with attentional deficits (Ros et al., 2022). These electrocortical impairments are commonly associated with reduced responses in the visual cortex to behaviourally relevant stimuli, poor motor planning, and impaired top-down control (Van Diepen et al., 2019). Accordingly, in line with the literature on ADHD patients reporting a linkage between EEG alpha power and behavioral impairments (Lenartowicz et al., 2018), our results add evidence to the notion that CUD patients may exhibit impaired attention and that this is mediated by anomalies in prefrontal activity. Further research should deepen our findings and examine any impact on other executive functions, e.g., by using other tasks specific for impulsivity.

These findings promisingly reveal a neurophysiological signature of cannabis abuse and indicate that EEG signals at specific frequencies may underpin distinctive behavioral impairments, consistent with our hypothesis. However, contrary to our hypothesis, the group-average number of errors in the Go/NoGo task did not reveal any significant attentional deficit overall, even though this effect was found and linked to the EEG anomalies when considering individual measures. Further research should examine whether deficits can be observed at the group level with other measures of task performance, such as trial-by-trial variability or time-dependent effects. In addition, EEG anomalies were obtained during spontaneous resting state and could be modified during an active attentional task to support normal behavioral performance.

## Limitations

The results of this study must be taken in the context of its limitations. Although the severity and ingestion of cannabis use was evaluated, in the absence of a standardized cannabis use unit we cannot control for the exact cannabis consumption and thus THC content (Hindocha et al., 2018; Prince et al., 2018). Participants had to abstain from cannabis 24 hours before the scheduled session to avoid the effects of acute intoxication, which leaves open the question whether the current results reflect the changes resulting from this short-term abstinence or continuous change due to cannabis use. Future studies should examine the differences between abstinence states (long-term and short-term) and acute intoxication. This is particularly important given the persistence of changes in dependent alcohol and heroin users (Winterer et al., 2003).

Other potential limitations are reflective of the imaging modality we used, i.e., EEG. Although EEG provides a direct measure of neural activity, it is most sensitive to sources within the cortical-mantle. Hence, our analyses were restricted to cortical network dynamics and did not allow for reliable assessment of subcortical structures which may have an important role in the control of attention (Gitelman et al., 1999; Vuilleumier, 2013).

Nevertheless, the results suggest a significant influence of cannabis on electrophysiological signals. Finally, it is important to note that the study’s design is unable to determine whether this is a cause or consequence of cannabis use. Longitudinal studies therefore should be performed to confirm whether these electrophysiological signals are consequences of cannabis use and not a physiological predisposition for its consumption.

## Conclusions

The present study examined for the first time the potential association between the electrocortical profile of cannabis abusers (i.e., spectral power) and their cognitive-behavioral performance. We confirmed previous work showing that cannabis users display statistically reduced cortical activation, indexed by excessive alpha power. This loss of “alpha desynchronization” suggests stronger inhibition of ongoing neuronal activity that may interrupt attentional processing. Importantly, we found that this abnormal EEG pattern predominates over frontal cortical regions and correlates with detection performance (i.e., hit rate) on an attentional task.

Thus, potentially treating cannabis abusers with therapies that decrease alpha rhythm production, such as neurofeedback, may be a promising approach for mitigating cognitive deficits and/or preventing drug relapse, as it is already used for improving attentional performance in ADHD (Ergenoglu et al., 2004).

## Supplementary Information

ESM 1 (DOCX 20 kb)

## Data Availability

The raw data supporting the conclusions of this article will be made available by the authors, without undue reservation.
