# Supplementary material for: Electrophysiological and behavioral correlates of cannabis use disorder
Source: Cogn Affect Behav Neurosci. 2022 Jun 13;22(6):1421–31. doi: 10.3758/s13415-022-01016-w (PMC9622528; doi:10.3758/s13415-022-01016-w)
Supplement: Supplementary file 1 — (DOCX 20 kb) [file 13415_2022_1016_MOESM1_ESM.docx]

**Supplemental data**

**Table 1.** Participant demographics (mean, *SD*)

|  | **Groups** | | |  |
| --- | --- | --- | --- | --- |
|  | **Controls** |  | **Patients CUD** | **P-value** |
| ***N*** | 24 |  | 24 | - |
| **Age** | 24.54 (4*.4*) |  | 26 (*5.39*) | 0.3123 |
| **Gender (F/M)** | 11F / 13M |  | 10F / 14M | - |
| **Formation** | High school degree / Bachelor |  | High school degree | 0.2512 |
| **CUD** | Absent |  | Present | - |
| **Number of meeting criteria for CUD** | 0.14 (*0.35*) |  | 6.17 (2*.78*) | < 0.0001 * |
| **Age at the first time** | - |  | 15.42 (2.50) |  |
| **Time (days) to smoke 1g Cannabis** | - |  | 2 (0*.91*) | - |
| **Weekly consumption (days/week)** | - |  | 6.29 (*1.23*) | - |
| **ASSIST** | 8.91 (*6.58*) |  | 51.12 (*18.20*) | < 0.0001* |
| Abbreviations: M/F, male/female; CUD, Cannabis Use Disorder | | | | |
